# Adjunctive antimycobacterial and immunomodulatory effects of *Allium cepa* (pink variety) in a rabbit model of latent-like mycobacterial infection

**DOI:** 10.3389/ftubr.2026.1874315

**Published:** 2026-08-06

**Authors:** Theophilus Kwesi Ninkyi, Eric Ogyam, Kelvin Atta Agyapong, Cynthia Amaning Danquah

**Affiliations:** Department of Pharmacology, Faculty of Pharmacy and Pharmaceutical Sciences, Kwame Nkrumah University of Science and Technology (KNUST), Kumasi, Ghana

**Keywords:** *Allium cepa*, latent tuberculosis infection, *mycobacterium aurum*, rabbit, tuberculosis

## Abstract

**Introduction:**

Latent tuberculosis infection (LTBI) represents a major global reservoir for active disease, characterized by persistent, metabolically inactive bacilli that are difficult to eradicate. Novel therapeutic approaches targeting bacterial persistence and host immune responses are needed.

**Methods:**

A rabbit model of latent-like mycobacterial infection was established using aerosolized *Mycobacterium aurum*. The rabbits were treated with isoniazid and rifapentine (HR), ethanolic extract of *Allium cepa* (EAC), and combination therapy. Treatment efficacy was evaluated using colony-forming unit (CFU) counts and haematological parameters (WBC, neutrophils, lymphocytes).

**Results:**

The CFU counts increased significantly during the induction phase, confirming successful establishment of the latent-like mycobacterial infection model, while stabilization of bacterial load over time suggested immune-mediated containment. During the treatment phase, significant reductions in CFU counts were observed in all treated groups compared with untreated controls (*p* < 0.0001), with the greatest reduction seen in combination therapy groups (HR + *Allium cepa* extract), indicating an improved efficacy relative to monotherapy. Haematological analysis revealed significant increases in WBC, neutrophils, and lymphocytes during infection, reflecting immune activation. Treatment resulted in normalization of WBC counts, reduction in neutrophilia, and stabilization of lymphocyte levels, indicating attenuation of inflammation and restoration of immune balance.

**Conclusion:**

*Allium cepa* demonstrated antimycobacterial and immunomodulatory effects in a rabbit model of latent-like mycobacterial infection and may have potential as an adjunctive therapeutic strategy in LTBI management.

## 1 Introduction

Latent Tuberculosis infection (LTBI) represents a critical yet often underappreciated component of the global tuberculosis burden. It is estimated that approximately one-quarter of the world's population is infected with *Mycobacterium tuberculosis* in a latent state, forming a vast reservoir from which active disease may emerge (1, 2). Unlike active tuberculosis, LTBI is characterized by the presence of viable bacilli contained within host tissues without overt clinical symptoms. However, this apparent equilibrium between pathogen and host immunity is unstable, and reactivation can occur, particularly under conditions of immune compromise such as HIV infection, malnutrition, or immunosuppressive therapy (2–4).

The pathophysiology of LTBI is complex and involves the formation of granulomatous structures that serve to contain the infection. Within these granulomas, *Mycobacterium tuberculosis* adopts a non-replicating or slowly replicating phenotype, enabling long-term survival under hypoxic and nutrient-limited conditions. These persistent bacilli exhibit phenotypic tolerance to many conventional anti-tuberculosis drugs, which are primarily active against rapidly dividing organisms. This intrinsic limitation of current chemotherapy contributes to the prolonged duration of LTBI treatment and the risk of incomplete bacterial eradication (2, 5, 6).

Current strategies for the management of LTBI rely heavily on preventive therapy using isoniazid or rifamycin-based regimens. While these regimens have demonstrated efficacy in reducing the risk of progression to active disease, their implementation is often limited by challenges such as long treatment duration, drug toxicity, poor adherence, and variable effectiveness across populations (7, 8). The World Health Organization has emphasized the importance of expanding LTBI treatment coverage, particularly among high-risk populations, but achieving this goal requires improved therapeutic strategies that are both effective and acceptable to patients (8–10).

The persistence of latent infection and the limitations of current treatment approaches highlight the need for novel therapeutic strategies that can target dormant bacilli and enhance host immune responses. In recent years, there has been increasing interest in host-directed therapies (HDTs), which aim to improve treatment outcomes by modulating the host immune response rather than directly targeting the pathogen. Such approaches are particularly relevant in LTBI, where immune mechanisms play a central role in maintaining bacterial containment (10, 11).

Natural products have historically been an important source of antimicrobial agents and continue to play a significant role in drug discovery. Medicinal plants, in particular, offer a diverse array of bioactive compounds with potential antimicrobial and immunomodulatory properties. Among these, the genus *Allium cepa* has attracted considerable attention due to its well-documented pharmacological activities, including antimicrobial, antioxidant, and anti-inflammatory effects (12, 13).

The biological activity of *Allium cepa* is largely attributed to its rich phytochemical composition, which includes flavonoids such as quercetin and kaempferol, as well as sulfur-containing compounds such as thiosulfinates (14, 15). These compounds have been shown to exert antimicrobial effects through multiple mechanisms, including disruption of microbial cell membranes, inhibition of enzyme systems, and interference with nucleic acid synthesis (16, 17). Additionally, flavonoids have been reported to possess immunomodulatory properties, which may enhance host defense mechanisms against infection (17, 18).

Several *in vitro* studies have demonstrated the antimycobacterial activity of *Allium cepa* extracts against surrogate organisms such as *Mycobacterium smegmatis* and *Mycobacterium aurum*, suggesting potential activity against *Mycobacterium tuberculosis* (19, 20). However, the translation of these findings into *in vivo* systems remains limited, particularly in the context of latent infection. The evaluation of plant extracts in animal models is essential for understanding their pharmacological effects within a complex biological system, including interactions with host immune responses.

The rabbit model of tuberculosis provides a valuable platform for studying both latent and active disease due to its ability to replicate key features of human tuberculosis, including granuloma formation and bacterial persistence (21, 22). This model allows for the assessment of therapeutic interventions in a physiologically relevant context, making it particularly suitable for evaluating therapeutic interventions in latent-like mycobacterial infection models (21, 23).

Therefore, the present study investigated the antimycobacterial and immunomodulatory effects of *Allium cepa* (pink variety) in a rabbit model of latent-like mycobacterial infection. Specifically, the study sought to evaluate its impact on bacterial persistence and haematological parameters, including WBC, neutrophil, and lymphocyte dynamics, with the broader goal of exploring its potential as an adjunctive therapeutic agent in LTBI management.

## 2 Materials and methods

### 2.1 Study design

This study was a controlled experimental *in vivo* investigation designed to evaluate the antimycobacterial and adjunctive therapeutic effects of the ethanolic extract of *Allium cepa* (pink variety) in a rabbit model of latent-like mycobacterial infection. The study assessed bacterial burden and haematological changes following treatment with standard latent tuberculosis preventive therapy, ethanolic extract of *Allium cepa* (EAC), and their combinations.

### 2.2 Experimental animals

Healthy adult New Zealand White rabbits ageing between 24 and 30 weeks, and weighing 3.5–5.0 Kg, were obtained from the Animal Experimental Unit of the Department of Pharmacology, Faculty of Pharmacy and Pharmaceutical Sciences, Kwame Nkrumah University of Science and Technology (KNUST), Kumasi, Ghana. The rabbits were housed in well-ventilated cages under standard laboratory conditions with a 12-hour light–dark cycle and had free access to standard rabbit feed and potable water *ad libitum*.

Animals were acclimatized for 7 days prior to commencement of the study. During acclimatization and throughout the experiment, rabbits were monitored daily for general health status, feed intake, grooming behaviour, posture, activity, and signs of respiratory distress. Humane endpoints included persistent anorexia, marked weight loss, severe respiratory compromise, or any condition likely to result in undue suffering.

All procedures involving animals were conducted in accordance with institutional guidelines for the care and use of laboratory animals and were approved by the Animal Research Ethics Committee (AREC) of KNUST. The study adhered to the principles of Replacement, Reduction, and Refinement (3Rs) in animal experimentation (24–26).

### 2.3 Preparation of ethanolic extract of *Allium cepa*

Fresh bulbs of *Allium cepa* (pink variety) were purchased from the Central Market, Kumasi, Ashanti Region, Ghana. The plant material was authenticated by Dr. George Henry Sam, Department of Herbal Medicine, KNUST, Kumasi, Ghana. A voucher specimen (KNUST/2019/HM1/013) was deposited at the Herbarium of the Department of Herbal Medicine, KNUST.

The bulbs were washed thoroughly, air-dried at room temperature, and pulverized into a coarse powder. The powdered material was extracted by maceration in 70% ethanol for 72 h with intermittent agitation. The resulting filtrate was concentrated under reduced pressure using a rotary evaporator at 40 °C to obtain the crude extract. The percentage yield of the extract was 12.4% (w/w). The extract was stored in sterile amber containers at 4 °C until used.

#### 2.3.1 Dose selection

The doses of 250, 500, and 1,000 mg/kg of the ethanolic extract of *Allium cepa* (EAC) were selected to evaluate potential dose-dependent antimycobacterial and immunomodulatory effects. Dose selection was based on previous experimental studies demonstrating the safety of *Allium cepa* extracts at comparable concentrations (27, 28) and on preliminary acute toxicity studies conducted in our laboratory, which indicated an oral LD_50_ greater than 5,000 mg/kg. The selected doses allowed evaluation of dose–response relationships while remaining well below the estimated toxic threshold. No overt signs of toxicity or treatment-related adverse effects were observed during the treatment period.

### 2.4 Sample size determination and group allocation

Sample size determination was guided by the resource equation approach and the longitudinal terminal-sampling design of the study. The primary outcome, lung colony-forming unit (CFU) counts, required scheduled sacrifice of animals at predefined time points. The resource equation method recommends an error degree of freedom between 10 and 20 to permit valid estimation of experimental error while avoiding unnecessary animal use.

A total of 73 rabbits were initially enrolled, comprising 63 infected rabbits and 10 naive rabbits. To confirm successful establishment of infection before treatment initiation, one rabbit from each infected group (7 groups) and two rabbits from the naive group were humanely sacrificed for baseline bacteriological assessment. This resulted in eight rabbits per experimental group for the treatment phase. This design ensured adequate sampling at each time point while minimizing animal use in accordance with the principle of reduction (Table 1).

**Table 1:** Experimental group allocation and sampling schedule for the LTBI model.

Group	Treatment	Initial number	Baseline sacrifice	Number at treatment initiation	Weekly sacrifice	Total treatment duration
1	Naïve Control	10	2	8	2/week	4 weeks
2	Negative control (infection only)	9	1	8	2/week	4 weeks
3	Infection + HR	9	1	8	2/week	4 weeks
4	Infection + 250 mg/kg EAC	9	1	8	2/week	4 weeks
5	Infection + 500 mg/kg EAC	9	1	8	2/week	4 weeks
6	Infection + 1,000 mg/kg EAC	9	1	8	2/week	4 weeks
7	Infection + HR + 500 mg/kg EAC	9	1	8	2/week	4 weeks
8	Infection + HR + 1,000 mg/kg EAC	9	1	8	2/week	4 weeks

HR comprised isoniazid and rifapentine administered at doses equivalent to standard preventive therapy regimens, with dosing based on an isoniazid content of 10 mg/kg. EAC, ethanolic extract of *Allium cepa*.

### 2.5 Induction and validation of the latent-like mycobacterial infection model

Rabbits were infected with *Mycobacterium aurum* (NCTC 10437) via aerosol exposure [inoculum concentration: 2.0 × 10^6^ colony-forming unit (CFU)/mL] using a laboratory aerosolization system under biosafety conditions. The aerosol challenge was designed to establish pulmonary infection while minimizing procedural stress to the animals. Following exposure, animals were returned to their housing units and monitored closely for clinical signs and general well-being throughout the induction period. The animals remained untreated for 5 weeks to permit immune-mediated containment of the infection.

To confirm successful establishment of infection, one rabbit from each infected group and two rabbits from the naïve control group were sacrificed during the induction phase. Lung tissues were aseptically collected for CFU determination, while blood samples were collected weekly for haematological analyses.

Chest radiographs were obtained at the end of the induction period to evaluate pulmonary involvement and exclude progressive active disease. Radiographs were examined for evidence of extensive pulmonary infiltrates, cavitation, consolidation, or severe pulmonary destruction suggestive of active tuberculosis. Animals demonstrating radiographic evidence of substantial lung damage were excluded from the latent-like cohort and reassigned to the active tuberculosis model. Representative radiographs from rabbits included in the latent-like cohort showed no evidence of extensive pulmonary pathology. These findings, together with stabilization of bacterial burden and absence of overt clinical deterioration, supported the characterization of the model as a latent-like mycobacterial infection state rather than progressive active disease.

### 2.6 Treatment protocol

Following confirmation of latent-like infection, rabbits received treatments according to their respective group allocations. Animals received either standard preventive therapy (HR), EAC at the allocated dose, or combination therapy. Treatments were administered daily for 4 weeks.

### 2.7 Anaesthesia and euthanasia

Rabbits selected for tissue collection were deeply anaesthetised using ketamine (35 mg/kg, intramuscularly) and xylazine (5 mg/kg, intramuscularly). Adequate depth of anaesthesia was confirmed by the absence of pedal withdrawal and corneal reflexes.

Euthanasia was subsequently performed by intravenous administration of sodium pentobarbital overdose (150 mg/kg). Death was confirmed by cessation of respiration and cardiac activity prior to tissue collection.

### 2.8 Sequential sampling and outcome measures

Two rabbits from each experimental group were humanely sacrificed weekly over a 4-week treatment period. This sequential sacrifice design enabled longitudinal assessment of treatment effects while maintaining adequate sample sizes at each time point.

Following euthanasia, lung tissues were harvested aseptically for CFU quantification. Blood samples were collected weekly for haematological analyses, including total white blood cell count, neutrophil count, and lymphocyte count.

For all invasive procedures, rabbits were anaesthetised using ketamine (35 mg/kg, IM) and xylazine (5 mg/kg, IM) to minimize pain and distress.

### 2.9 Statistical analysis

Data was analysed using GraphPad Prism software (GraphPad Software, San Diego, CA, USA). Results were expressed as mean ± standard error of the mean (SEM). Differences between groups and time points were evaluated using two-way analysis of variance (ANOVA). When significant effects were observed, Sidak's multiple comparison test was used for *post hoc* analyses.

Interaction terms were interpreted as indicating that the effect of time differed according to treatment group rather than simply reflecting temporal changes within groups. Statistical significance was established at *p* < 0.05.

## 3 Results

### 3.1 Validation of the latent-like mycobacterial infection model

#### 3.1.1 Colony-forming unit (CFU) counts during the induction phase

The CFU counts increased significantly in the infected rabbits compared with the non-infected controls over the infection period. The increase was evident as early as 24 h post-infection and remained elevated at 5 weeks, indicating successful establishment of infection in the latent-like mycobacterial infection model. The relative decline in CFU counts over the 5-week period despite no treatment supports the establishment of a latent-like infection state, as the defense system of the rabbit was able to keep the infection under control without increase in CFU count.

Inferential Analysis: Two-way ANOVA followed by Sidak's *post-hoc* test showed that infection group significantly affected CFU counts [*F*_(1,8)_ = 1,067, *p* < 0.0001], indicating that temporal changes differed according to infection status. However, the interaction between infection group and time was not significant (*p* = 0.0611), indicating that although infected animals had higher bacterial loads than controls, the pattern of increase over time was relatively consistent between groups and supports the idea that the rabbit immune system was able to keep the infection under control over the time period without any external intervention.

#### 3.1.2 Radiographic assessment of the latent-like infection model

These figures are representative chest radiographs of naive control rabbits and rabbits included in the latent-like infection cohort following aerosol exposure to *Mycobacterium aurum* (rabbits 1 and 2 part of the uninfected group, and 3, 4, 7, and 8 were part of the infected group). Radiographs from the latent-like cohort showed no evidence of extensive pulmonary infiltrates, cavitary lesions, diffuse consolidation, or severe pulmonary destruction suggestive of progressive active disease. Animals demonstrating marked radiographic abnormalities were excluded from the latent-like infection model and reassigned to the active tuberculosis cohort. These radiographic findings, together with stabilization of pulmonary bacterial burden and the absence of overt clinical deterioration, supported the characterization of the infection as a latent-like mycobacterial state.

#### 3.1.3 Haematological changes during the induction phase

##### 3.1.3.1 Total white blood cell (WBC) count

Total white blood cell counts increased following infection. The increase was modest at 24 h but became significantly elevated by 5 weeks, suggesting progressive activation of systemic immune responses.

Inferential Analysis: Two-way repeated measures ANOVA demonstrated a significant interaction between infection status and time (*p* = 0.0001), indicating that the pattern of change in WBC counts over time differed between infected and non-infected rabbits. Specifically, infected rabbits exhibited a progressive increase in WBC counts from 24 h to 5 weeks post-infection, whereas WBC counts remained relatively stable in non-infected controls. To further explore these differences, Sidak's *post hoc* test revealed:

✓ 0 h vs. control: not significant (*p* = 0.6637) (meaning no significant difference between the WBC counts of the infected group and the non-infected group at 0-hour).
✓ 24 h vs. control: significant increase (*p* = 0.0060) (meaning there is a significant difference between the WBC of the infected group and the non-infected group at 24-hour)
✓ 5 weeks vs. control: significant increase (*p* = 0.0114) (meaning there is a significant difference between the WBC of the infected group and the non-infected group at 5 weeks).

This suggests immune activation becomes apparent after infection progression.

##### 3.1.3.2 Neutrophils count

Neutrophil counts increased significantly during the infection period, particularly at later time points, indicating activation of the innate immune response.

Inferential Analysis: Two-way repeated measures ANOVA revealed a significant interaction between infection status and time (*p* = 0.0376), indicating that temporal changes in neutrophil counts differed between infected and non-infected rabbits. Infected rabbits showed a progressive increase in neutrophil counts from 24 h to 5 weeks post-infection, whereas counts remained relatively stable in non-infected controls. The main effect of time was not significant (*p* = 0.2429), suggesting that neutrophil responses were driven predominantly by infection status rather than time alone.

To further support the analysis a Sidak *post-hoc* test revealed:

✓ 0 and 24 h vs. control: not significant (*p* = 0.9951 and *p* = 0.1841 respectively) (meaning no significant difference between the neutrophil counts of the infected group and the non-infected group at 0- and 24-hour).
✓ 5 weeks vs. control: significant increase (*p* = 0.0112) (meaning there is a significant difference between the neutrophil counts of the infected group and the non-infected group at 5 weeks).

##### 3.1.3.3 Lymphocytes count

Lymphocyte levels increased gradually following infection, indicating activation of adaptive immune responses during the establishment of latent-like mycobacterial infection.

Inferential Analysis: Two-way repeated measures ANOVA revealed a significant interaction between infection status and time (*p* = 0.0238), indicating that the pattern of change in lymphocyte counts over time differed between infected and non-infected rabbits. Specifically, lymphocyte counts in infected rabbits changed differently across the observation period compared with those in non-infected controls. However, the main effect of time was not statistically significant (*p* = 0.0638), suggesting that changes in lymphocyte counts were not attributable to the passage of time alone. These findings indicate that lymphocyte responses were influenced primarily by infection status and its interaction with time.

To further support the analysis a Sidak *post-hoc* test revealed:

✓ 0 and 24 h vs. control: not significant (*p* = 0.9993 and *p* = 0.4017 respectively) (meaning no significant difference between the lymphocyte counts of the infected group and the non-infected group at 0- and 24-hour).
✓ 5 weeks vs. control: showed a strong increase (*p* ≈ 0.0577, trend toward significance)

This suggests that lymphocyte changes were mainly driven by infection status rather than time alone.

### 3.2 Effects of treatment on pulmonary bacterial burden

During the treatment phase, CFU counts declined significantly in treated groups compared with untreated controls. This reduction indicates that the administered therapy effectively suppressed bacterial replication in the LTBI rabbit model.

Inferential Analysis: Two-way ANOVA revealed a significant treatment effect on CFU counts (*p* < 0.0001). Dunnett's *post-hoc* comparisons showed that treated animals exhibited significantly lower bacterial loads compared with untreated infected controls, indicating therapeutic efficacy, with the mean difference of the HR + 1,000 mg/kg vs. negative control (480, 95% CI = 472.9–487.1) being the closest to the mean difference of the naïve group vs. negative control (495, 95% CI = 487.9–502.1) followed by standard LTBI drugs (HR) (472.0, 95% CI = 464.9–479.1). The 1,000 mg/kg dose had a mean difference of 467.0 (95% CI = 459.9–474.1), and the least mean difference was 332.5 (95% CI = 325–339.6) for the 250 mg/kg dose of extract. This shows that though both the HR and the 1,000 mg/kg had individual effects on the CFU count when used separately, their combination produced greater reductions in CFU counts than either intervention alone, suggesting improved therapeutic efficacy with adjunctive administration.

### 3.3 Effects of treatment on haematological parameters

#### 3.3.1 Total white blood cell (WBC) count

Total WBC counts decreased during treatment, suggesting a reduction in systemic inflammation as bacterial burden declined. Treated animals exhibited values closer to baseline compared with untreated controls.

Inferential Analysis: Two-way ANOVA analysis followed by Dunnett's *post-hoc* demonstrated significant differences between treatment groups (*p* < 0.0001). These findings suggest that treatment modulated systemic immune responses associated with infection.

#### 3.3.2 Neutrophil count

Neutrophil counts decreased during the treatment phase, indicating reduced inflammatory signaling as bacterial loads were suppressed.

Inferential Analysis: Two-way ANOVA showed significant differences among treatment groups (*p* < 0.0001), suggesting that treatment influenced neutrophil dynamics and reduced innate immune activation.

#### 3.3.3 Lymphocyte count

Lymphocyte counts gradually normalized during treatment. This pattern suggests stabilization of adaptive immune responses as antigenic stimulation decreased due to bacterial clearance.

Inferential Analysis: ANOVA revealed significant treatment-dependent differences in lymphocyte levels (*p* = 0.0001). *Post-hoc* comparisons indicated normalization of lymphocyte counts in treated animals.

## 4 Discussion

The present study provides a comprehensive evaluation of the antimycobacterial and immunomodulatory effects of *Allium cepa* in a rabbit model of latent-like mycobacterial infection. By integrating bacteriological and haematological outcomes across both the induction and treatment phases, the study offers important insights into the dual role of the extract in modulating bacterial persistence and host immune responses. The findings not only confirm the biological activity of *A. cepa in vivo* but also extend previous *in vitro* observations into a physiologically relevant disease model.

**Figure 1:**
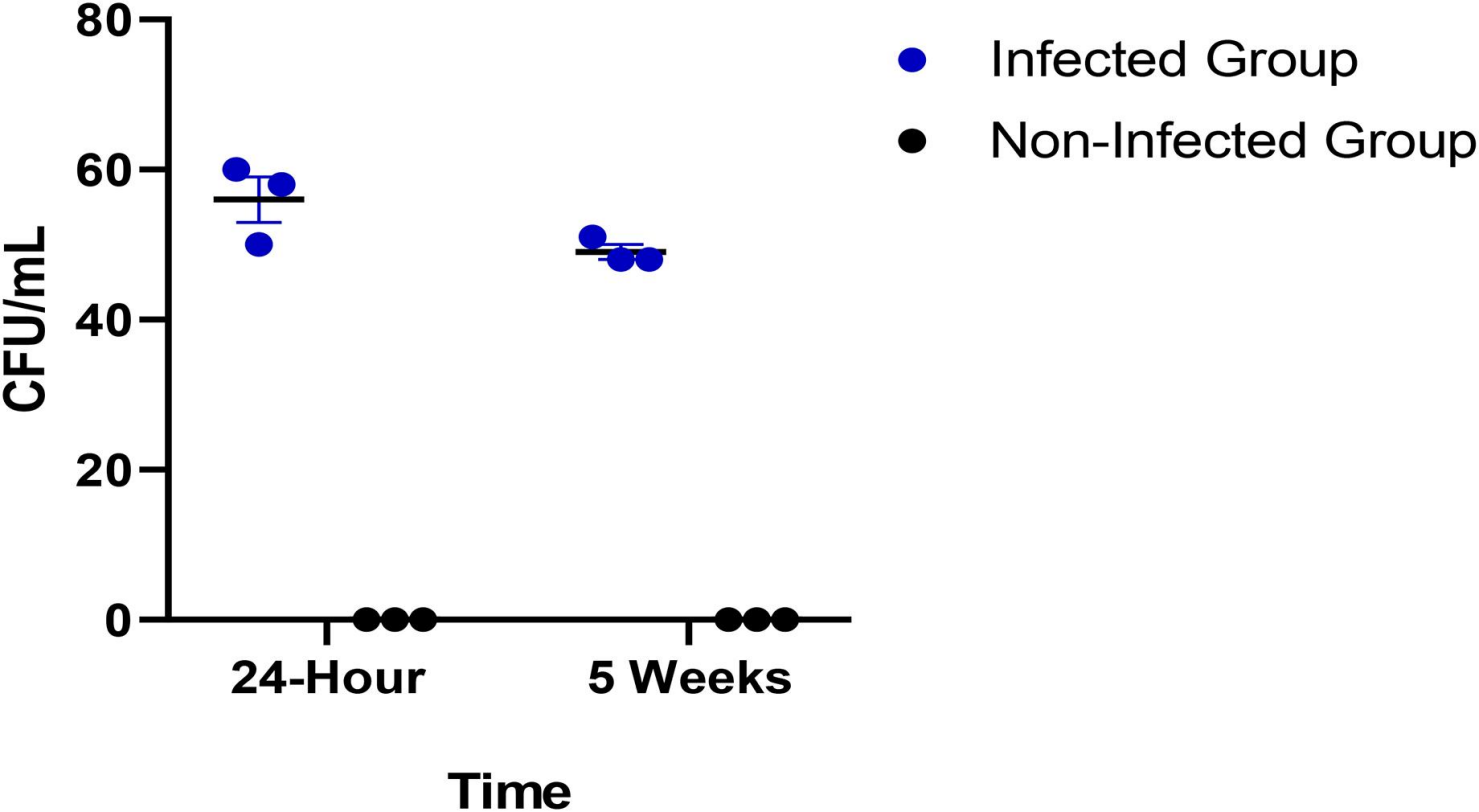
CFU count during induction phase.

A critical first step in interpreting the therapeutic effects of the extract is the successful establishment of the LTBI model. As demonstrated in Figure 1, CFU counts increased significantly in infected animals compared to non-infected controls, confirming successful infection. However, the observed stabilization and slight decline in CFU counts over time, despite the absence of treatment, suggest that the host immune system was able to partially contain bacterial proliferation. This pattern is consistent with a latent-like infection state characterized by immune-mediated containment of bacterial proliferation (29, 30). The lack of a significant interaction between time and infection status further supports the notion that bacterial growth remained controlled rather than progressive, thereby validating the experimental model used in this study.

**Figure 2:**
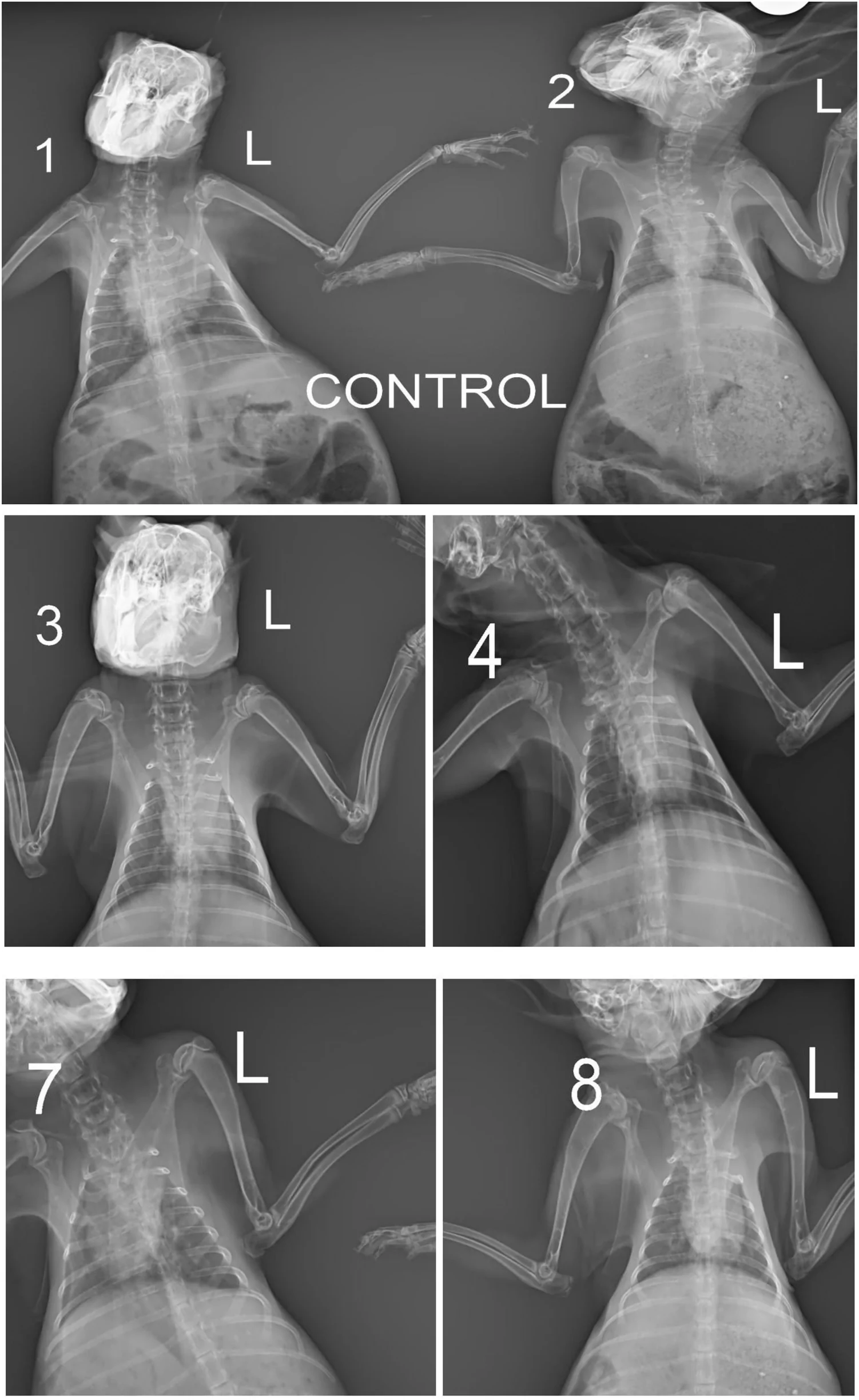
Representative thoracic radiographs (L, left side; numbers, numbers allocated to each rabbit).

Radiographic assessment provided an additional layer of validation for the latent-like mycobacterial infection model. As illustrated in Figure 2, representative thoracic radiographs obtained from rabbits included in the latent-like cohort showed no evidence of extensive pulmonary infiltrates, cavitary lesions, diffuse consolidation, or severe pulmonary destruction typically associated with progressive active tuberculosis. In contrast to active disease, where radiographic abnormalities often reflect extensive tissue involvement and ongoing bacterial replication, the absence of marked pulmonary pathology in these animals supports the notion of immune-mediated containment of infection. Importantly, animals demonstrating substantial radiographic abnormalities were excluded from the latent-like cohort and reassigned to the active tuberculosis model. Although radiographic findings alone cannot definitively establish latency, their integration with bacteriological stabilization and the absence of overt clinical deterioration strengthens the characterization of this model as a latent-like mycobacterial infection state rather than uncontrolled active disease. These findings address an important limitation of relying solely on CFU dynamics for model validation and underscore the value of incorporating multimodal assessments when studying persistent mycobacterial infections.

**Figure 3:**
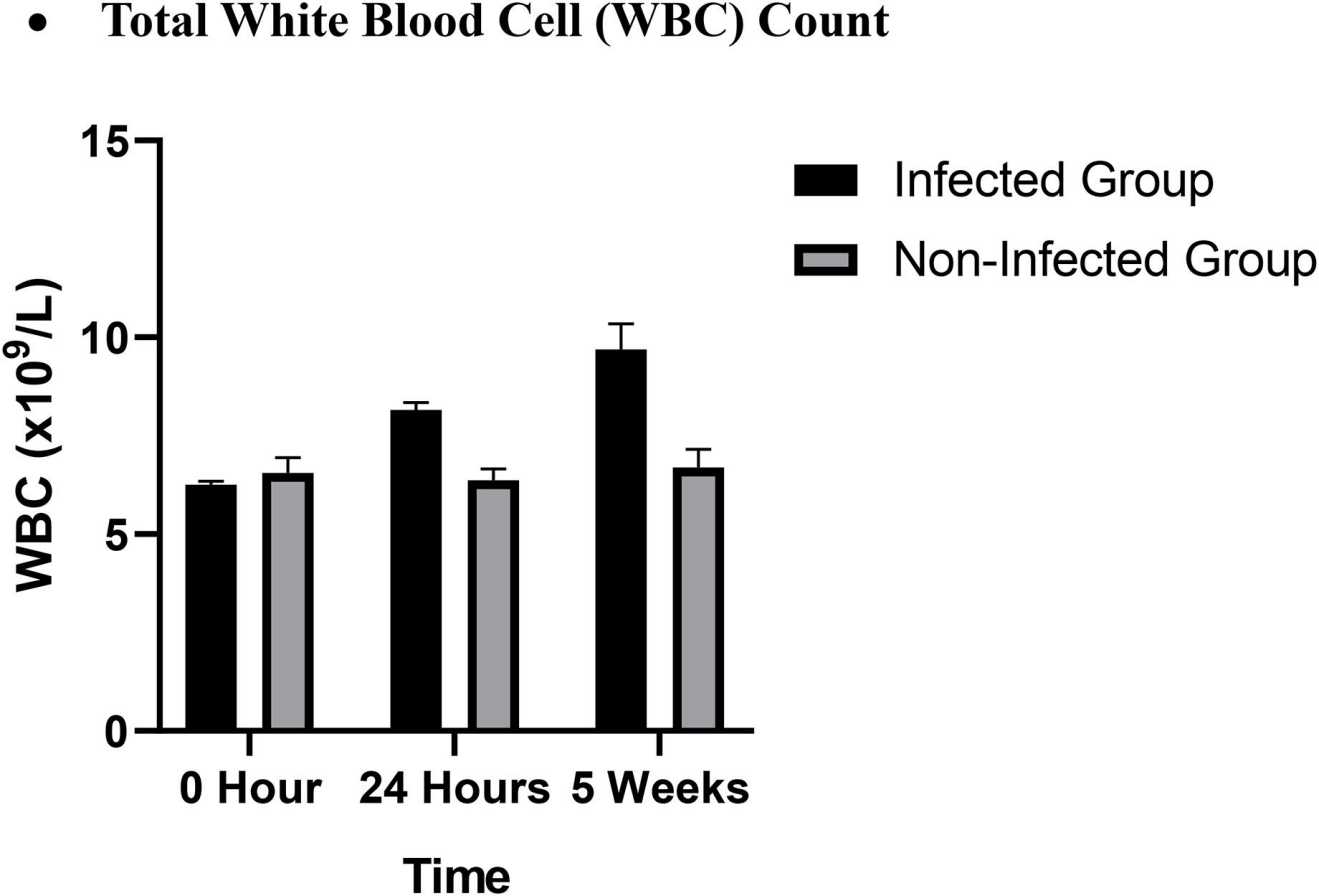
WBC count during induction phase.

**Figure 4:**
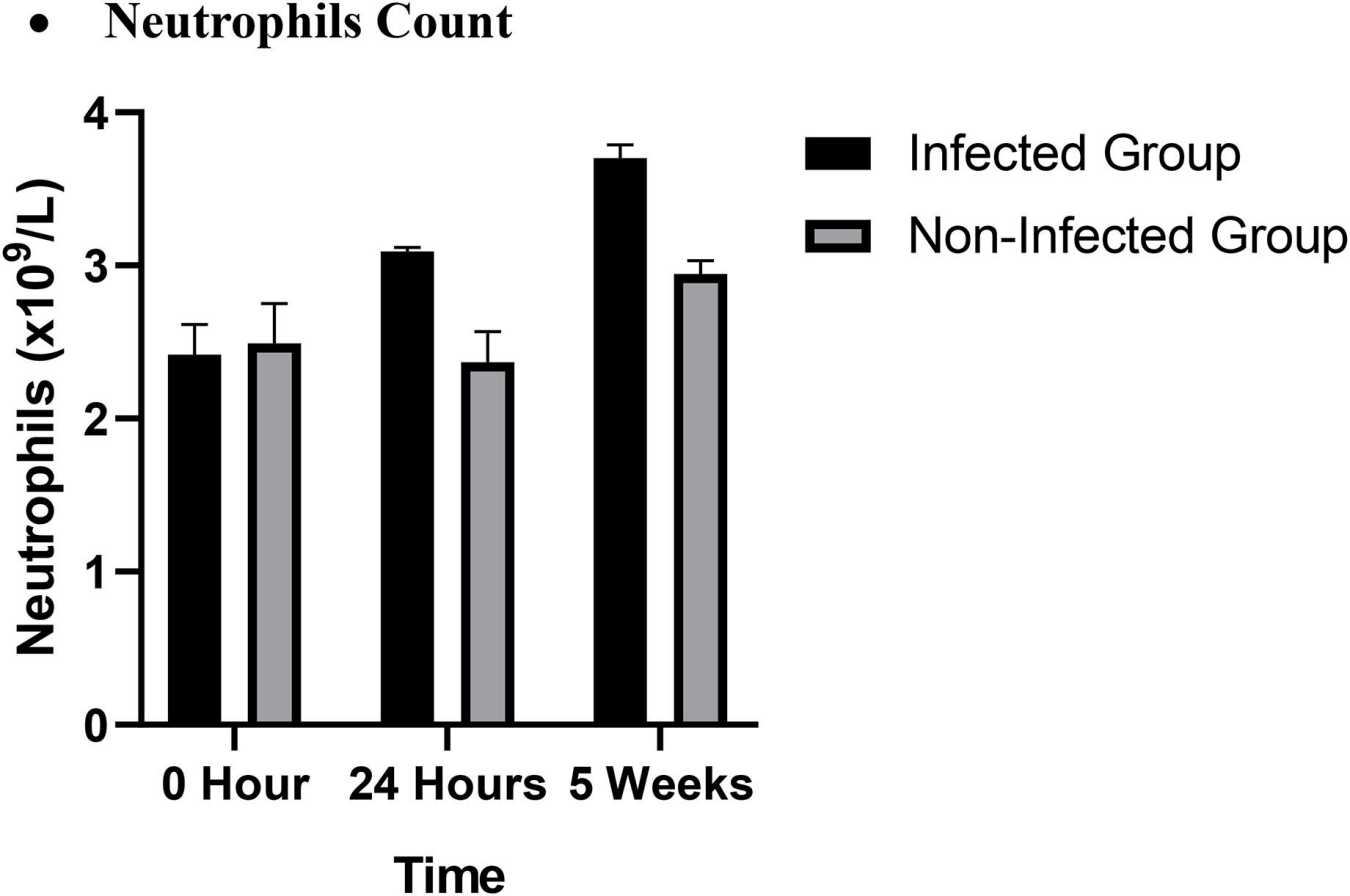
Neutrophils count during induction phase.

The haematological changes observed during the induction phase provide additional evidence of host immune activation. As shown in Figure 3, total WBC counts increased progressively following infection, with statistically significant elevations at later time points. This trend reflects systemic immune activation in response to mycobacterial exposure, consistent with previous reports that leukocytosis is a hallmark of infection-induced immune responses (31, 32). Similarly, the significant increase in neutrophil counts observed in Figure 4, particularly at 5 weeks post-infection, indicates activation of the innate immune system. Neutrophils play a crucial role in early defense against *Mycobacterium* species; however, excessive neutrophil activity has been associated with tissue damage and disease progression in tuberculosis (33, 34).

**Figure 5:**
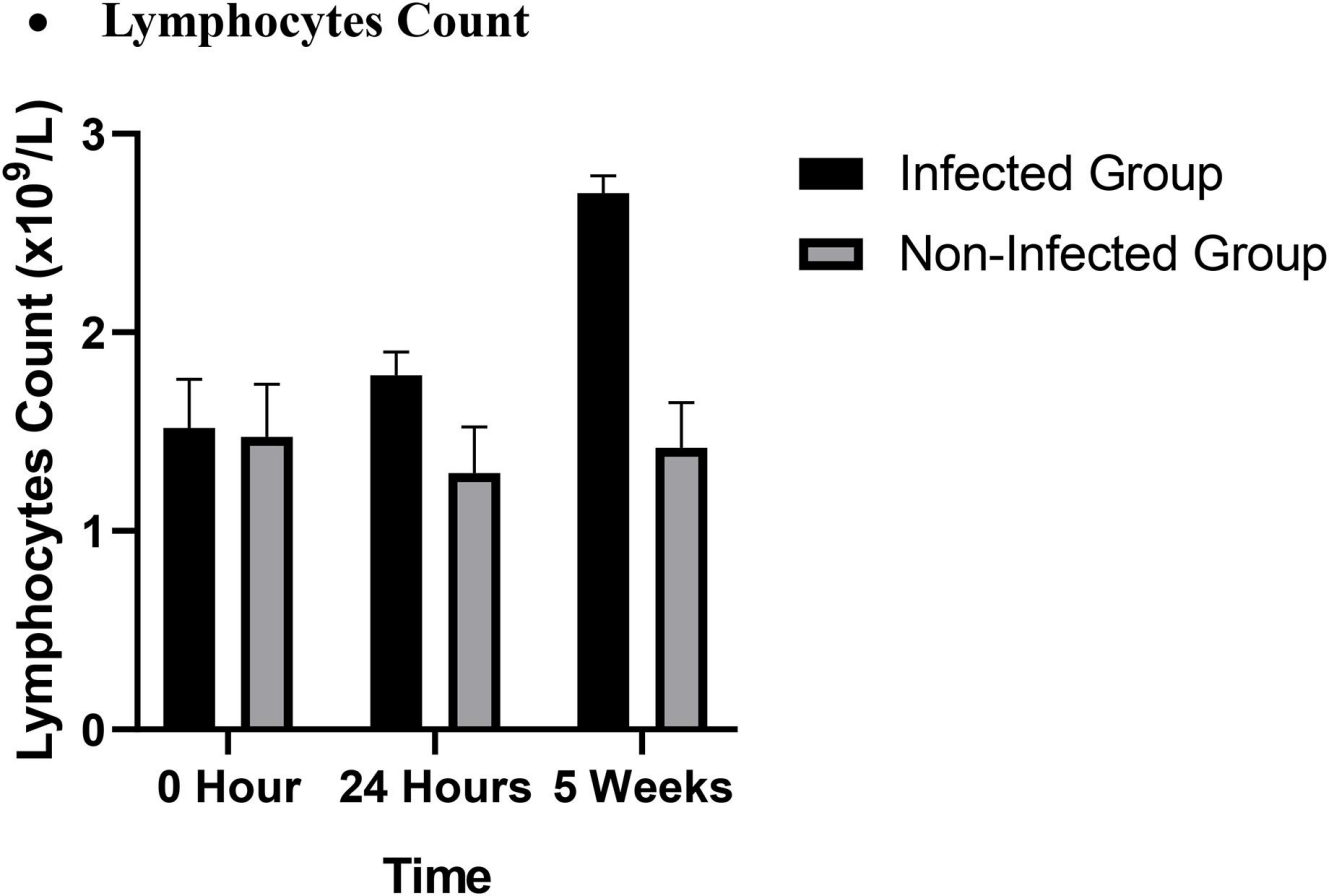
Lymphocytes count during induction phase.

In contrast, lymphocyte dynamics, as illustrated in Figure 5, showed a gradual increase during the induction phase, reflecting activation of adaptive immune responses. Although the changes did not reach strong statistical significance at all time points, the trend toward increased lymphocyte counts suggests possible activation of adaptive immune responses, which is essential for the containment of latent infection (35, 36). Together, these findings suggest that the model reproduces several immunological features associated with latent-like mycobacterial infection, including balanced innate and adaptive immune responses.

**Figure 6:**
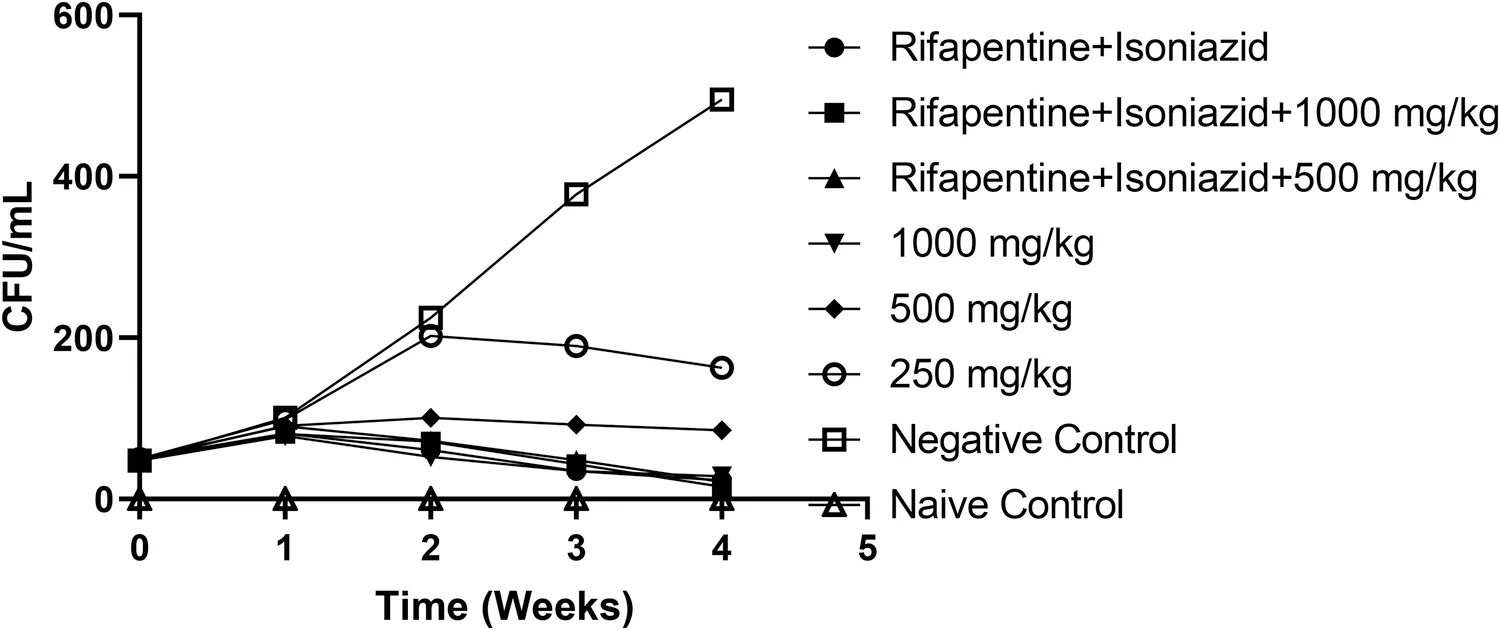
Longitudinal changes in CFU counts during treatment.

During the treatment phase, the impact of *Allium cepa* extract on bacterial load becomes particularly evident. As shown in Figure 6, CFU counts declined significantly in treated groups compared to untreated controls, with the most pronounced reductions observed in groups receiving combination therapy (HR + extract). The inferential analysis confirms a highly significant treatment effect (*p* < 0.0001), indicating that the observed reductions are not due to chance. Notably, the combination of HR and high-dose extract (1,000 mg/kg) produced bacterial reductions comparable to the naïve group, suggesting near-complete bacterial clearance. This finding is particularly important in the context of LTBI, where persistent bacilli exhibit tolerance to conventional drugs that primarily target actively replicating organisms (37, 38).

The improved outcomes observed in combination therapy groups suggest that adjunctive administration of *A. cepa* and standard anti-tuberculosis drugs may enhance therapeutic efficacy relative to monotherapy. Such adjunctive effect has been widely reported for plant-derived compounds, which may enhance drug uptake, inhibit resistance mechanisms such as efflux pumps, or target complementary metabolic pathways (39, 40). In this study, the superior performance of combination therapy compared to monotherapy strongly supports the potential of *A. cepa* as an adjunctive therapeutic agent.

**Figure 7:**
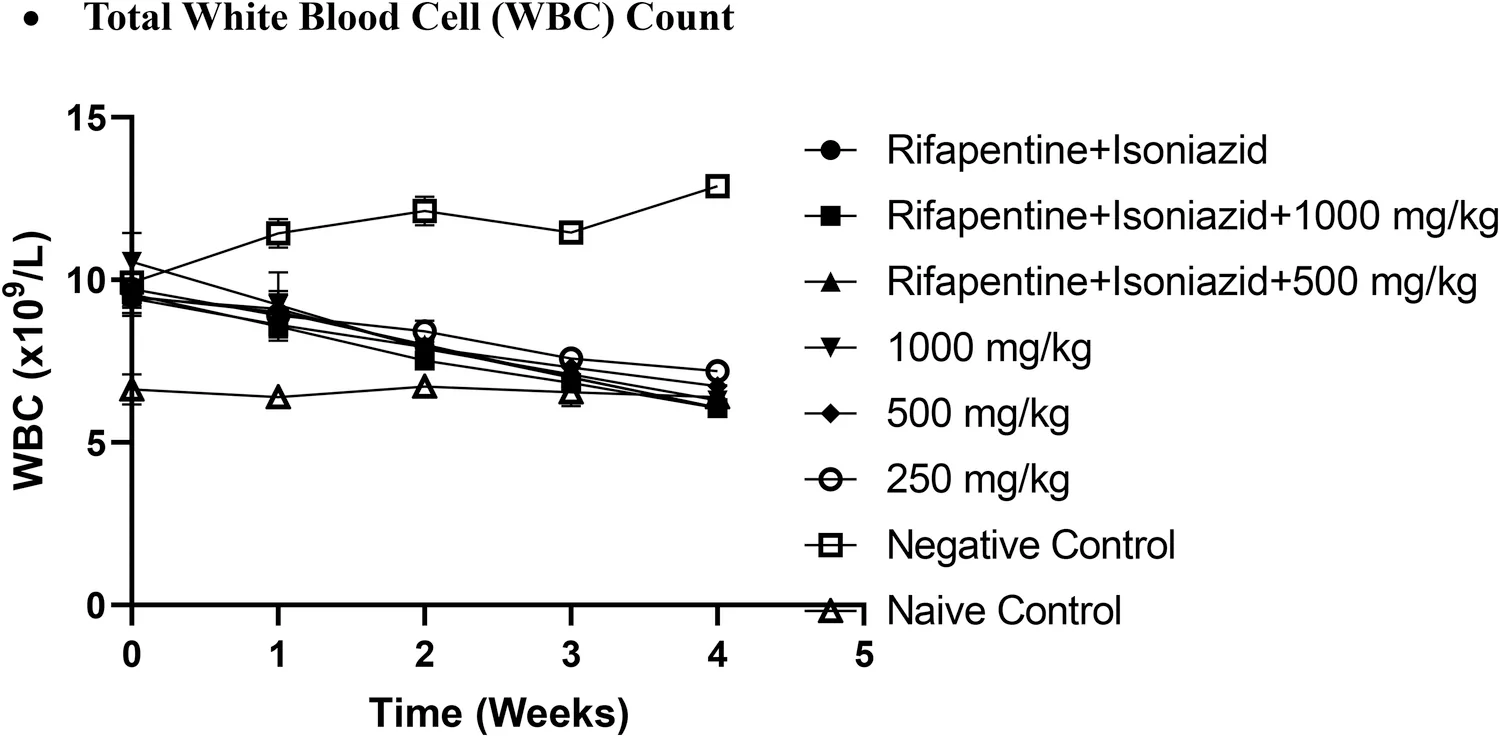
WBC count during treatment phase.

The immunomodulatory effects of the extract are further reflected in the normalization of haematological parameters during treatment. As shown in Figure 7, WBC counts declined significantly in treated groups, approaching baseline levels observed in non-infected animals. This reduction suggests resolution of infection-associated inflammation and restoration of immune homeostasis. Chronic immune activation is a defining feature of tuberculosis, and its resolution is essential for preventing tissue damage and disease progression (41, 42).

**Figure 8:**
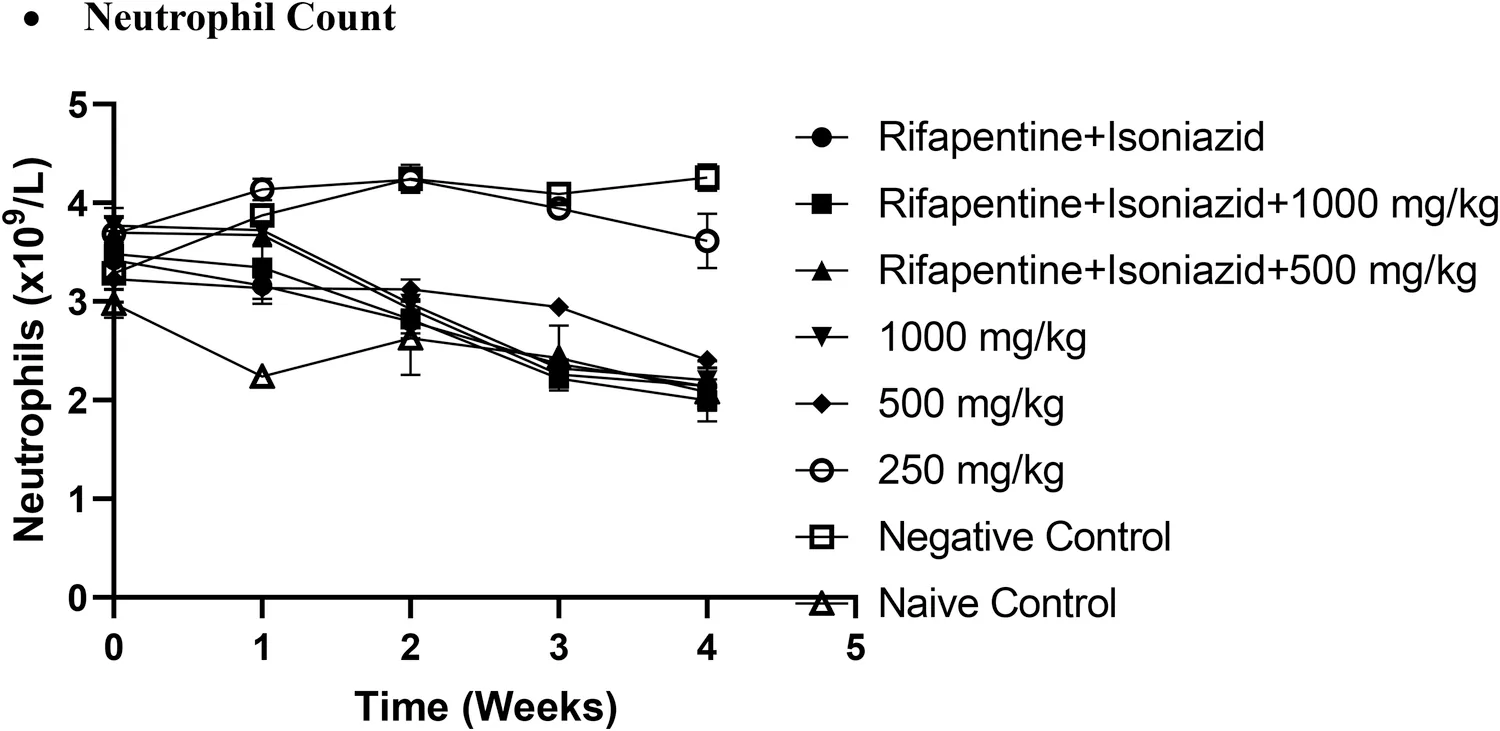
Neutrophils count during treatment phase.

Similarly, neutrophil counts decreased significantly during treatment, as illustrated in Figure 8. This reduction indicates attenuation of excessive innate immune activation, which is beneficial in preventing host tissue damage. Excessive neutrophil activity has been linked to poor outcomes in tuberculosis due to the release of proteolytic enzymes and reactive oxygen species (43, 44). Therefore, the ability of *A. cepa* to modulate neutrophil responses represents an important therapeutic advantage.

**Figure 9:**
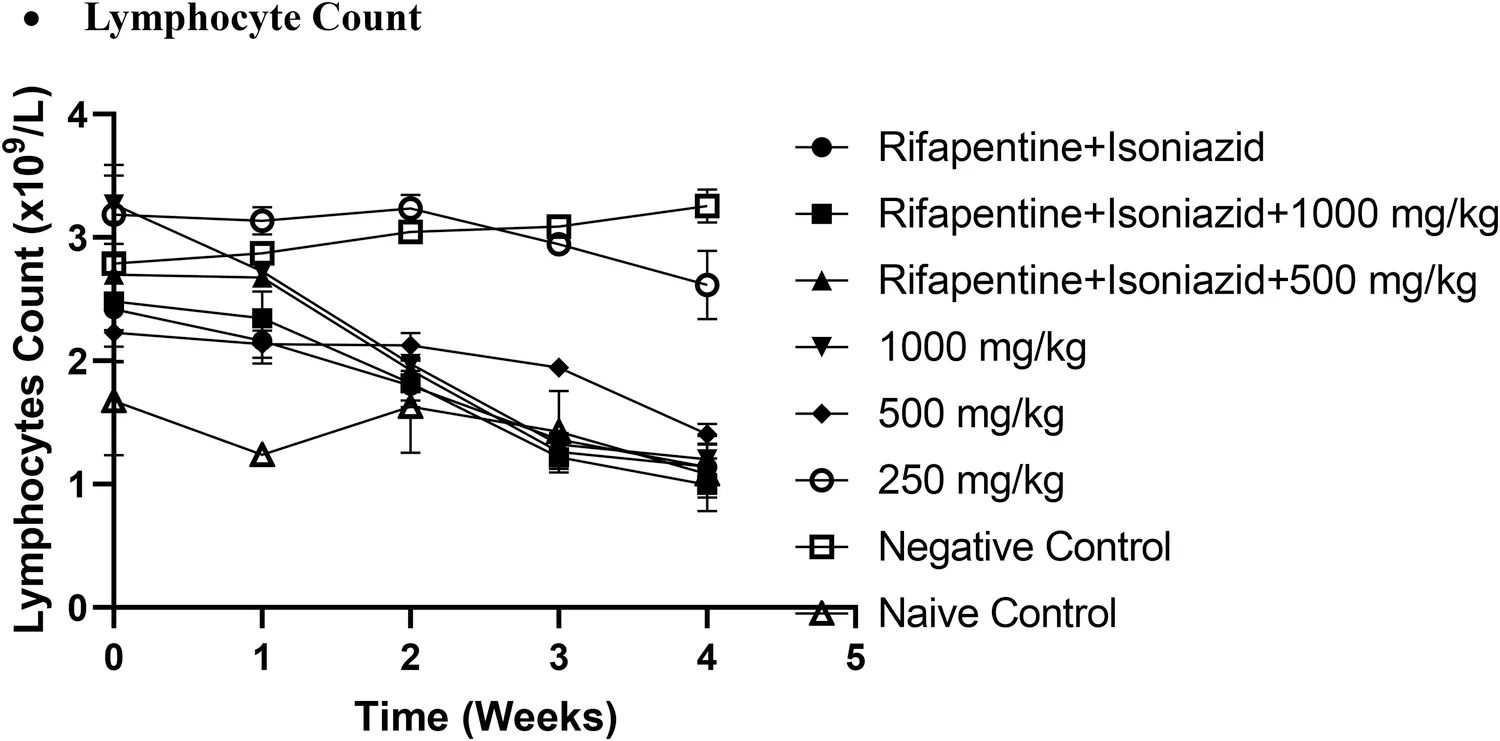
Lymphocytes count during treatment phase.

In contrast, lymphocyte counts, as shown in Figure 9, gradually normalized during treatment, reflecting stabilization of adaptive immune responses. This pattern suggests that as bacterial load decreases, antigenic stimulation is reduced, allowing immune responses to return to a balanced state. The restoration of lymphocyte function is critical for maintaining long-term control of infection and preventing reactivation (35, 36).

The dual antimicrobial and immunomodulatory effects observed in this study align closely with the concept of host-directed therapy (HDT). HDTs aim to enhance host immune responses while simultaneously targeting pathogen survival, thereby improving treatment outcomes (45, 46). In the context of LTBI, where immune regulation plays a central role in maintaining bacterial containment, such approaches are particularly relevant.

The observed biological effects can be attributed to the phytochemical composition of *Allium cepa*. Flavonoids such as quercetin have been shown to possess both antimicrobial and anti-inflammatory properties, while sulfur-containing compounds such as thiosulfinates can disrupt microbial enzyme systems and metabolic pathways (14, 15). These compounds may act adjunctively to inhibit bacterial survival while modulating host immune responses. In addition, previous *in vitro* findings from this research demonstrated anti-efflux and antibiofilm activities, which may further enhance bacterial susceptibility and reduce persistence. Efflux pump inhibition may increase intracellular drug concentrations, while antibiofilm activity may disrupt protective bacterial niches, both of which are critical in targeting latent infection.

Importantly, the findings of this study bridge the gap between *in vitro* observations and *in vivo* efficacy. While previous studies have demonstrated the antimycobacterial activity of *A. cepa in vitro* (17, 18), the present study provides direct evidence of its effectiveness in an animal model that closely mimics human disease. The rabbit model is particularly valuable due to its ability to replicate granuloma formation and bacterial persistence, key features of tuberculosis pathogenesis (20).

Despite these promising findings, certain limitations should be acknowledged. The use of *Mycobacterium aurum* as a surrogate organism does not fully replicate the pathogenicity of *Mycobacterium tuberculosis*. Additionally, the study did not include molecular analyses of immune responses, such as cytokine profiling, which would provide deeper mechanistic insights. Future studies should address these limitations and explore the pharmacokinetics, toxicity, and clinical applicability of the extract.

In summary, this study demonstrates that *Allium cepa* exerts significant antimycobacterial and immunomodulatory effects in a rabbit model of latent tuberculosis. The extract reduces bacterial load, enhances the efficacy of standard therapy, and modulates key immune parameters, supporting its potential as an adjunctive therapeutic agent.

## 5 Conclusion

The findings of this study indicate that the ethanolic extract of *Allium cepa* (pink variety) possesses promising adjunctive potential in the management of latent tuberculosis. Treatment with the extract was associated with reduced mycobacterial burden and favourable modulation of host immune responses, particularly when used alongside standard anti-tuberculosis therapy. These results support the growing interest in plant-derived agents as complementary approaches for tuberculosis control. However, further studies are required to identify the bioactive constituents, elucidate their mechanisms of action, and confirm their efficacy and safety against *Mycobacterium tuberculosis* in advanced preclinical and clinical studies.

## Data Availability

The raw data supporting the conclusions of this article will be made available by the authors, without undue reservation.
